# Impact of Cigarette Smoking on Outcome of Hepatocellular Carcinoma after Surgery in Patients with Hepatitis B

**DOI:** 10.1371/journal.pone.0085077

**Published:** 2014-01-15

**Authors:** Xu-Feng Zhang, Tao Wei, Xue-Min Liu, Chang Liu, Yi Lv

**Affiliations:** Department of Hepatobiliary Surgery, the First Affiliated Hospital of Medical College, Xi'an Jiaotong University, Xi'an, China; Kaohsiung Medical University Hospital, Kaohsiung Medical University, Taiwan

## Abstract

**Background and Objectives:**

Cigarette smoking is a potential risk factor for hepatocellular carcinoma (HCC) initiation, partially through interaction with hepatitis B virus (HBV). We examined the hypothesis that cigarette smoking might be associated with HBV-related HCC recurrence and patient survival after curative surgery.

**Patients and Methods:**

Data of 302 patients with HBV infection who had undergone curative resection for HCC were prospectively collected from 2008 to 2011. Smoking status and smoking quantity (pack-years, PY) were asked at admission. Factors affecting recurrence-free survival (RFS) were examined. RFS and liver-specific mortality (LSM) stratified by risk factors were compared with log-rank test.

**Results:**

109 were current smokers. Current smokers were not different from non-smokers in tumor burden and surgical procedure. Univariate and multivariate analysis identified that heavy smoking (PY ≥20) was the most significant factor associated with HBV-related HCC recurrence after curative surgical resection (*p* = 0.001), followed by anti-HBV treatment (*p*<0.01), current smoking (*p* = 0.028), surgical margin <1 cm (*p* = 0.048) and blood transfusion >600 ml (*p* = 0.028). The median RFS in non-smokers, ex-smokers and current smokers was 34 months, 24 months and 26 months, respectively (*p* = 0.033). Current smokers had significantly worse RFS rate and increased 5-year cumulative LSM than non-smokers (*p* = 0.024, and *p*<0.001, respectively). Heavy smokers had significantly worse RFS than non- and light smokers (0<PY<20) (*p*<0.001, respectively) and higher cumulative LSM than non-smokers and light smokers (*p* = 0.003 and 0.001, respectively). Furthermore, in current smokers, continuing smoking postoperatively was strongly associated with poorer RFS and higher LSM than those who quit smoking postoperatively (*p* = 0.016 and *p* = 0.003, respectively).

**Conclusions:**

Smoking history and quantity appears to be risk factors for HBV-related HCC recurrence and LSM of patients after surgery. For smokers, continued smoking postoperatively might accelerate tumor recurrence and patient death. Therefore, smoking abstinence should be strongly recommended to patients pre- and postoperatively.

## Introduction

Hepatocellular carcinoma (HCC) is the fifth leading cause of cancer-related death all over the world and responsible for more than 700 000 death annually [1]. It was estimated that, in 2002, 82% of liver cancer cases occurred in developing countries, with 55% in China alone[1]. In China, HCC ranks the second most common cause of cancer-related death, accounting for 370 000 death each year[2]. And the major causes of HCC in high-risk and low-risk populations are different. In endemic area such as China and Africa, chronic infection of hepatitis B virus (HBV) is the main risk factor. However, in low-risk area such as western countries, chronic alcohol consumption and hepatitis C virus (HCV) are important risk factors[3]–[5].

Cigarette smoking is associated with HCC development, independent of geography and race-ethnicity[5]–[7]. Cigarette carries over 4000 toxic substances which causes hazardous adverse effects on almost every organ in the body. Scientists used to focus on the harmful effects of smoking on direct contacting organs, such as lung, oral, larynx, esophagus, stomach, etc. However, recent studies identified that smoking causes a variety of adverse effects on organs that have no direct contact with smoke itself such as liver. Liver is a major organ for the metabolism and transformation of more than 40 tobacco-related active compounds. Several constituents of cigarette smoke are known liver carcinogens in humans and animal models, e.g. 4-aminobiphenyl, arsenic and vinyl chloride, etc.[6]–[10]. However, the effect of cigarette smoking on HCC biology remains obscure.

Preoperative smoking cessation is always recommended to patients by surgeons because of potential increase of postoperative complications related[11], [12]. However, whether cigarette smoking has any long term impact on tumor recurrence and prognosis of patients is not well defined. In some smoking-related cancers, tobacco use is associated with tumor recurrence, including prostate carcinoma, bladder cancer, colon and gastric cancer[13]–[16]. Recently, Shih *et al.* [17] found a negative impact of smoking on overall survival of the patients with viral hepatitis-related HCC. However, the significance of cigarette smoking on HCC recurrence after curative resection has not yet been investigated. Although interaction between cigarette smoking and HCV infection on the risk of HCC has been well documented, the possible association between cigarette smoking and HBV infection on the risk of HCC is still controversial, implying different mechanism of cigarette smoking in promoting HCC development with HBV and HCV background[18].

The present prospective study enrolled HCC patients with HBV infection who underwent surgical resection in a large tertiary hospital. The impact of pre- and post-operative smoking habit on recurrence-free survival (RFS) and liver-specific mortality (LSM) of patients with HBV-related HCC was investigated.

## Patients and Methods

### Patients

Data of 397 patients undergoing curative resection was collected prospectively in our hospital from January 2008 to January 2011. Amongst, 10 cases with hepatitis C, 3 with dual hepatitis B and C and 82 with no evidence of hepatitis virus infection were excluded, and the remaining 302 cases identified hepatitis B serologically were enrolled. Patients and their relatives were informed with written consent documents for the data collection and study at admission and during follow up. The study was approved by the patients and their relatives during follow-up. And also the research was approved by the Ethics Committees of the First Affiliated Hospital of Medical College, Xi'an Jiaotong Univerisity. The diagnosis of HCC was made by computed tomography (CT) scan, ultrasonography, magnetic resonance image and/or angiography preoperatively, and confirmed by pathological examination postoperatively. All resected tumors were examined pathologically for the degree of differentiation of HCC, macroscopic vascular invasion, surgical margin, tumor number and maximum size.

### Treatments and Follow-up

Hepatic resection was the treatment of patients with HCC when available based on the general condition, tumor status, preoperative liver function, as well as the future remnant liver parenchymal. Curative resection was defined as complete excision of the tumor with tumor-free surgical margins.Anti-HBV medications were prescribed by our hepatologists, and patients were selected for anti-HBV treatment mainly according to the guidelines devised by the American Association for the Study of Liver Disease [19], [20]. The medications for chronic hepatitis B were interferon-α (IFNα) and nucleostide analogues, including lamivudine, entecavir, tenofovir disoproxil fumarate, adefovir dipivoxil, and telbivudine.

After discharge, all patients were followed up every 3–6 months by upper abdominal CT as routine, and also serum alpha-fetoprotein (AFP) for the patients in the first 3 years and then every 6 months or 1 year afterwards. HBV status and HBV DNA were also regularly examined for evaluation of efficiency of antiviral treatment by our hepatologists. If tumor recurrence was confirmed, repeated resection, radiofrequency ablation (RA), and/or transcatheter arterial chemoembolization or infusion (TACE/TAI) were administered when available. The recurrence-free survival time was defined as the time between the date of surgery and the date of recurrence confirmed by examination. Liver-specific death was defined as that patients died of HCC recurrence or metastasis, viral hepatitis, or end-staged liver disease and the train of events initiated, such as variceal bleeding, hepatic encephalopathy, HBV-related renal failure, etc.

### Measurement of exposure to tobacco and alcohol

Biochemical testing data was recorded before the surgery. Patients were asked if they smoke regularly and, if so, at what age they started, numbers of cigarettes smoked per day (average), and how many years of smoking. Smoking data included pack-years smoked (PY) [[the average number of packages of cigarettes smoked per day multiplied by the number of years smoked]. Tobacco exposure was characterized as none (<100 cigarettes during their lifetime), light smoker (PY>0 and <20) and heavy smoker (PY≥20) [21]–[23]. Smoking status was defined as non-smoker, ex-smoker and current smoker. A regular smoker was defined as one who smokes at least 1 cigarette per day for over 1 year and a current smoker as one who regularly smokes for at least 1 year and continues to smoke within 1 year prior to surgery, an ex-smoker was defined as one who quit smoking for more than 1 year before surgery [17], [24]. Postoperative smoker was defined as one who smokes within first year postoperatively for at least one year or till death. In accordance with previous guidelines, we categorized alcohol drinking/abuse into never drinker, low-moderate (1–50 g/day) and medium-high drinker (>50 g/day) [25]. Smokers and alcohol abusers were routinely recommended to give up the bad habit both preoperatively and postoperatively.

### Statistic analysis

Data were expressed as mean ± standard error (S.D.) for numerical variables or percentages for nominal variables. Mann-Whitney U test or *t* tests were used to compare numerical variables, and the Chi-Square test or Fisher's exact test was carried out to compare nominal variables between the groups. The overall and disease-free survivals were calculated by the Kaplan-Meier method, and the differences in survival between groups were compared using the log-rank test. Impact factors for the recurrence of HCC after hepatic resection were analyzed by univariate analysis firstly, and those with *p* value less than 0.05 were enrolled for multivariate analysis using Cox proportional hazards model. Statistical analysis was carried out using SPSS 17.0. *p*<0.05 was considered statistically significant.

## Results

### Comparison of baseline characteristics

Of the 302 patients with hepatitis B (253 men and 49 women, with a mean age of 48.9 yr) enrolled and analyzed, 109 and 25 patients were documented as current smokers and ex-smokers, respectively, while 168 patients had no severe cigarette smoking history. The characteristics of the patients in the three groups were shown in table 1. No significant differences were noted between the two groups in age, gender and coexistence of liver cirrhosis. Cigarette smoking patients tended to have concomitant alcohol abuse than non-smokers (*p*<0.001). Although more patients with liver function of Child-Pugh classification B were identified in current smokers than non-smokers (*p* = 0.005), biochemical tests of alanine aminotransferase (ALT), aspartate aminotransferase (AST), total bilirubin (TBIL) and albumin level were not different statistically between the two groups. In addition, smokers had higher hemoglobin but lower platelet count, and prolonged prothrombin time than non-smokers (*p*<0.05, respectively). No significant differences were observed between current and non-smokers in tumor size, number, vascular invasion, pathological stage and preoperative AFP level. But ex-smokers seemed having more multiple lesions and higher frequency of vascular invasion than non- and current smokers (*p*<0.05, respectively). The surgery-related variables in terms of surgical mode, margin and perioperative blood transfusion were not significantly different between the three groups. However, smokers had longer hospital stay than non-smokers (*p* = 0.007).

**Table 1:** Clinicopathological characteristics of the patients with HBV-realted HCC.

Clinical parameters	Non-smokers (n = 168)	Ex-smokers (n = 25)	Current Smokers (n = 109)	*p* value
	NO. or mean	NO. or mean	NO. or mean	
Age (y)	48 (±1)	52(±2)	49 (±1)	0.44
Gender (M/F)	134/34	21/4	98/11	0.08
Alcohol drinking	15 (8.9%)	8 (32%)	50 (45.9%)	<0.001*
Liver cirrhosis	136 (81.0%)	21 (84%)	96 (88.1%)	0.29
Child-pugh classcification (A/B/C)	163/5/0	22/3/0	95/14/0	0.005*
Hemoglobin (g/L)	126.0 (±1.3)	132.7 (±3.8)	132.9 (±1.7)	0.004*
Platelet count (X10^9^/L)	134.9 (±6.0)	100.2 (8.7)	106.6 (±5.3)	0.001*
ALT (U/L)	49.2 (±2.6)	52.2 (±3.3)	55.3 (±3.1)	0.57
AST (U/L)	59.8 (±2.7)	56.5 (±3.5)	58.9 (±4.8)	0.93
Total bilirubin (umol/L)	17.6 (±0.6)	89.5 (±39.5)	25.4 (±3.5)	<0.001
Albumin(g/L)	39.4 (±0.4)	38.6 (±1.4)	38.2 (±0.5)	0.15
Prothrombin time (s)	12.8 (±0.1)	13.1 (0.4)	13.4 (±0.2)	0.023*
Alpha-fetoprotein (ng/mL)	9957 (±1942)	15273 (±11262)	9569.8 (±3731)	0.74
Maximum tumor size (cm)	6.1 (±0.3)	6.1 (±0.5)	5.9 (±0.3)	0.96
tumor number (≥2)	32 (19.0%)	13 (52%)	19 (17.4%)	<0.001
Vascular invasion	17 (10.1%)	7 (28%)	17 (15.6%)	0.038
Differentiation degree (poor/moderate/well)	9/116/43	1/13/11	4/77/28	0.36
Surgical mode				0.40
anatomical	118 (70.2%)	17 (68%)	68 (62.4%)	
nonanatomical	50 (29.8%)	8 (32%)	41 (37.6%)	
Surgical margin≥1 cm	119 (70.8%)	18 (72%)	79 (72.5%)	0.96
Blood transfusion (mL)	548.2 (±51.7)	424.0 (±164.2)	627.5 (±64.8)	0.36
Hospital stay (d)	22.7 (±0.6)	24.5 (±0.7)	27.6 (±2.0)	0.007*

Percentage or standard error in parentheses. HBV, hepatitis B virus; HCC, hepatocellular carcinoma; ALT, alanine aminotransferase; AST, aspartate aminotransferase. * *p*<0.05 between current and non-smokers.

### Preoperative smoking is a risk factor promoting tumor recurrence after surgical treatment

By May 2013, the median follow-up time was 26 months (1–62 months). HCC recurred after surgical resection in 197 patients with hepatitis B (65.2%). To investigate the risk factors contributing HCC recurrence, we enrolled 22 potential variables and analyzed by univariate analysis (Table 2). Univariate analysis identified the following seven variables as risk factors promoting HCC recurrence in patients with hepatitis B: antiviral treatment (*p*<0.001), smoking status (*p* = 0.033), smoking pack-years (*p*<0.001), AFP higher than 20 ng/mL (*p* = 0.018), tumor size larger than 5 cm (*p* = 0.005), multiple tumor lesions (*p* = 0.042), surgical margin less than 1 cm (*p* = 0.048) and perioperative blood transfusion quantity more than 600 mL (*p* = 0.004). When introducing the eight variables with *p* value lower than 0.05 in univariate analysis into multivariate analysis using Cox proportional hazards model, and as a result, we identified smoking status (current against non-smokers, risk 1.4, 95% CI 1.1–1.7, *p* = 0.028), smoking PY ≥20 (risk 2.1, 95% CI 1.3–3.3, *p* = 0.001), No antiviral treatment (risk 2.0, 95% CI 1.5–2.8, *p*<0.001), surgical margin less than 1 cm (risk 1.4, 95% CI 1.0–2.1, *p* = 0.048) and blood transfusion more than 600 mL (risk 1.4, 95% CI 1.0–2.0, *p* = 0.028) were independent risk factors contributing HCC recurrence in patients with hepatitis B (Table 3).

**Table 2:** Univariate Analysis of Risk Factors for Recurrence of HBV-related HCC after Surgical Resection.

Variables	n	Number of recurrence	HR (95% CI)	Median Survival (m)	*p* value
Age (≤40/>40 yr)	86/216	54/143	1.0 (0.7–1.4)	30.0/30.0	0.99
Gender (male/female)	253/49	164/33	1.1 (0.7–1.6)	36.9/29.5	0.21
HBeAg (+/−)	178/124	119/78	0.9 (0.7–1.2)	28.6/32.5	0.16
HBV DNA (copies/mL) (<103/≥103)	155/76*	109/32	1.1 (0.9–1.3)	33.3/30.4	0.12
Antiviral treatment (Yes/No)	148/127**	83/90	1.8 (1.3–2.4)	36.3/26.6	<0.001
Alcohol drinking					
Never/low/heavy drinker	229/32/41	153/18/26	1.0 (0.7–1.5)	30.3/29.5/28.9	0.91
Smoking status			1.2 (1.1–1.5)		0.033
Non-smoker	168	95		33.8	
Ex-smoker	25	20		23.6	
Current smoker	109	82		25.9	
Smoking pack-years			1.4 (1.2–1.7)		<0.001
= 0	168	95		33.9	
>0 and <20	64	44		31.8	
≥20	70	58		19.0	
Liver cirrhosis (+/−)	253/49	162/35	1.0 (0.7–1.4)	30.2/29.2	0.79
Child-Pugh grade (A/B)	280/22	179/18	1.2 (0.8–2.0)	30.5/24.0	0.40
Hemoglobin (<110/≥110 g/L)	50/252	34/163	0.9 (0.6–1.3)	30.5/27.6	0.56
Platelet count (X10^9^/L) (<100/≥100)	137/165	87/110	1.0 (0.7–1.3)	30.4/29.2	0.87
Total bilirubin (≤17.1/>17.1 umol/L)	156/146	100/97	1.1 (0.8–1.5)	28.5/29.2	0.41
Albumin(<35/≥35 g/L)	77/225	54/143	1.0 (0.7–1.4)	26.2/30.4	0.87
Prothrombin time (<14/≥14 s)	220/82	165/32	1.2 (0.9–1.5)	30.1/28.0	0.13
Alpha-fetoprotein (<20/≥20 ng/mL)	90/212	54/143	1.4 (1.1–2.0)	35.0/27.5	0.018
Tumor size (<5/≥5 cm)	120/182	70/127	1.5 (1.1–2.0)	34.9/26.5	0.005
Tumor number (1/≥2)	238/64	150/47	1.4 (1.0–1.9)	31.5/24.9	0.042
Capsule formation (+/−)	69/233	146/51	1.3 (0.9–1.7)	31.2/26.7	0.16
Vascular invasion (+/−)	41/261	28/169	1.2 (0.8–1.8)	27.1/30.5	0.37
Surgical margin (<1/≥1 cm)	86/216	49/148	0.7 (0.5–1.0)	27.7/34.6	0.048
Blood transfusion (≤600/>600 mL)	197/105	118/79	1.5 (1.0–2.0)	33.0/24.5	0.004

CI, confidence interval; HR, hazard ratio; HBV, hepatitis B virus; HCC, hepatocellular carcinoma.* Data of serum level of HBV DNA in 71 patients were available; ** Data of antiviral treatment in 27 patients were available.

**Table 3:** Mutivariate Analysis of Risk Factors for Recurrence of HBV-related HCC after Surgical Resection.

Variables	Hazard Ratio	95% CI	*p* value
Smoking status (Current)	1.4	1.1–1.7	0.028
Smoking pack-years (≥20)	2.1	1.3–3.3	0.001
Anti-HBV treatment	2.0	1.5–2.8	<0.001
Tumor size (≥5 cm)	1.3	0.9–1.8	0.13
Tumor number (≥2)	1.3	0.9–1.8	0.18
Surgical margin (<1 cm)	1.4	1.0–2.1	0.048
Blood transfusion (>600 mL)	1.4	1.0–2.0	0.028
Alpha-fetoprotein (≥20 ng/mL)	1.4	1.0–2.0	0.91

HBV, hepatitis B virus; HCC, hepatocellular carcinoma; CI, confidence interval.

### Impact of smoking status on prognosis of HCC with hepatitis B after surgical treatment

**Figure 1:**
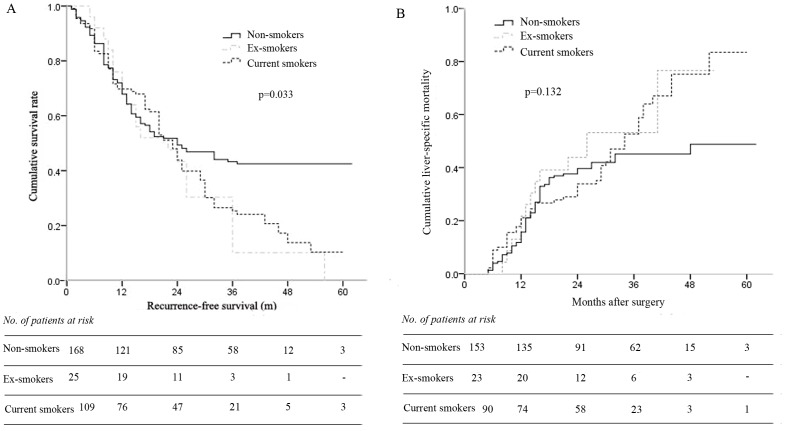
Kaplan-Meier curves for survival of non-smokers, ex-smokers and current smokers. (A), recurrence-free survival; (B), cumulative liver-specific mortality.

Figure 1A shows the Kaplan-Meier curves for RFS of non-, ex- and current smokers. The median RFS in non-smokers, ex-smokers and current smokers was 34 months, 24 months and 26 months, respectively (*p* = 0.033). It was identified that current smokers had significantly worse RFS and 5-year RFS rate than non-smokers (*p* = 0.024 and *p*<0.001). Although no significant difference in RFS was documented between ex-smokers and non-smokers (*p* = 0.102), after five years' follow up, none of the 25 ex-smokers survived, compared with a 5-year RFS of 42% in non-smokers (*p*<0.001).

During follow up, 15 patients in non-smokers, 2 in ex-smokers and 19 in current smokers were died of non-liver-related causes, such as heart attack, pulmonary disease, diabetes, intracerebral hemorrhage, etc. These patients were then excluded for further overall survival analysis. Figure 1B shows the Kaplan-Meier curves for cumulative LSM of non-, ex- and current smokers. The median overall survival in non-smokers, ex-smokers and current smokers was 40 months, 30 months and 33 months, respectively (*p* = 0.132) Although there was no significant difference of overall survival among the three groups, the 5-year cumulative LSM after surgery was significantly higher in current smokers when compared to non-smokers (Figure 1B, 86% *vs* 49%, *p*<0.001).

### Impact of smoking pack-years on prognosis of HCC with hepatitis B after surgical treatment

**Figure 2:**
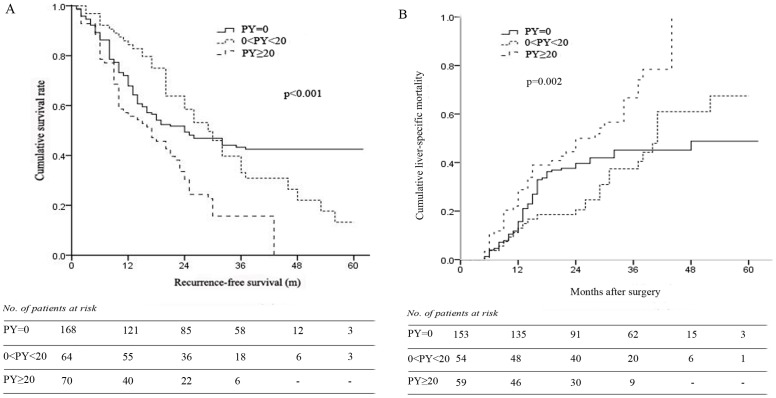
Kaplan-Meier curves for survival of non-smokers, light smokers (pack-years >0 and <20) and heavy smokers (pack-years≥20). (A), recurrence-free survival; (B), cumulative liver-specific mortality.

Kaplan-Meier curves for RFS and LSM of non-smokers (PY = 0), light smokers (0<PY<20) and heavy smokers (PY≥20) were plotted in figure 2 A and B, respectively. The median RFS of non-smokers, light smokers and heavy smokers were 34 months, 32 months and 19 months, indicating that the RFS was significantly worse in heavy smokers than in non- and light smokers (*p*<0.001, respectively) but not different between non- and light smokers (*p* = 0.721). However, the 5-year RFS rate in light smokers was significantly lower than that in non-smokers (figure 2A, 42% vs. 13%, *p*<0.001). The median overall survival of non-smokers, light smokers and heavy smokers were 40 months, 40 months and 26 months, showing decreased overall survival rates in heavy smokers compared to non- and light smokers (*p* = 0.003 and 0.001, respectively, figure 2B). The 1-, 3- and 5-year cumulative LSM were 21%, 45% and 49% in non-smokers, 15%, 41% and 71% in light smokers, and 29%, 75% and available in heavy smokers (*p* = 0.002).

### Impact of postoperative smoking on prognosis of HCC with hepatitis B

**Figure 3:**
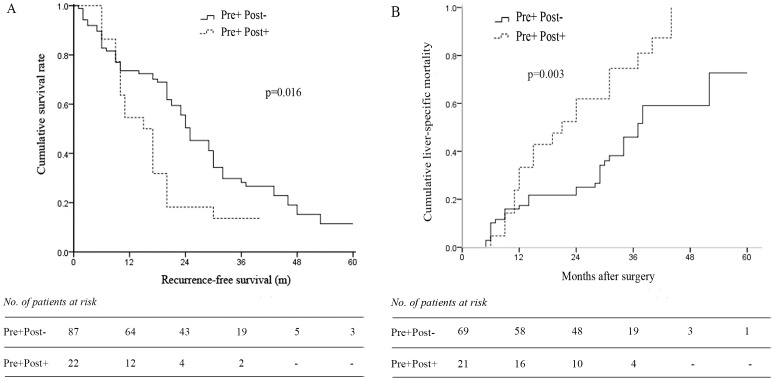
Kaplan-Meier curves for survival of patients who smoked preoperatively but quit postoperatively (Pre+ Post−) and patients who smoked preoperatively and continued postoperatively (Pre+ Post+). (A), recurrence-free survival; (B), cumulative liver-specific mortality.

Very few patients of non-smokers and former smokers would possibly smoke postoperatively. We further investigated possible influence of postoperative smoking on prognosis of patients. As defined, 87 patients of current smokers quit smoke postoperatively (Pre+ Post−), while 22 patients continued smoking postoperatively (Pre+ Post+). As shown in figure 3 A and B, patients of Pre+ Post+ had significantly poorer RFS and increased LSM than those of Pre+ Post− (*p* = 0.016 and *p* = 0.003, respectively). The 1- and 3-year cumulative LSM were 17% and 52% in Pre+ Post−, and 33% and 80% in Pre+ Post+. At the end time of follow-up, 63 (72%) patients of Pre+ Post− and 19 (86%) patients of Pre+ Post+ were identified tumor recurrence or distal metastasis. 54 and 14 patients in the two groups had undergone re-treatments after tumor recurrence, respectively, including re-resection, radiofrequency ablation,and/or transcatheter arterial chemoembolization, etc.

## Discussion

In the present study, univariate and multivariate analysis identified preoperative cigarette smoking status and smoking pack-years as independent risk factors contributing to recurrence of HCC with hepatitis B background after surgery. This finding implied that cigarette smoking might promote HCC recurrence even after curative resection. To our best knowledge, this is the first study evaluating the influence of cigarette smoking on HCC recurrence after surgery. A recent comprehensive study analyzed 2,273 patients (1990 with HBV or HCV and 283 without), and demonstrated a deleterious effect of cigarette smoking on overall survival of the patients with viral hepatitis-related HCC[17]. Unfortunately, this study did not take clinical treatment of HCC into consideration or evaluate any correlation between cigarette smoking and HCC recurrence. Because of high prevalence of HBV but relatively low HCV infection rate in Chinese population, we enrolled 302 patients HBV-related HCC. The most significant difference between the study from Shih *et al.* [17] and ours is that our study included patients who had curative resection only, since some patients with unresectable tumors or intolerable liver function tended to have much more comorbidity and extremely poor prognosis.

Notably, heavy smokers with smoking PY≥20 appeared to be associated with tumor recurrence and patient death. In addition, both heavy and light smokers had decreased 5-year RFS but increased LSM than non-smokers. This finding implied that cigarette smoking might contribute to tumor recurrence and patient death at a dose-dependent manner, which, however, could not be fully demonstrated in the present study due to short term follow up and small number of patients enrolled. It has been demonstrated that cigarette smoking increases risk of HCC development at a dose- and duration-dependent manner[5], [23]. Shih *et al.* proved in their study that smoking PY≥10 was a cut-off value associated with increased HCC death than non-smokers[17]. However, some other studies available proved that patients with smoking PY>0 or ≥50 had significantly higher incidence of gastric or bladder cancer recurrence after radical resection[15], [16]. Therefore, the severity of smoking attributing to recurrence of different tumors might be different in amount.

Current smokers had higher proportion of concomitant alcohol abuse than non- & former smokers. Although it was recognized as a synergistic factor with smoking for HCC occurrence[23] and a negative prognostic factor for HCC survival[17], to our best knowledge, there is no study investigating potential influence of alcohol drinking on HCC recurrence after surgery. In the present study, we also enrolled “alcohol drinking” as a potential variables influencing HCC recurrence. However, we did not find any difference of recurrence-free survival among never drinker, low and heavy drinkers, which implying alcohol intake seems not to be an independent risk factor of HCC recurrence. However, It can not be ignored that only a small sample of alcohol drinkers were enrolled in our study since alcohol drinking is not that severe and popular among Chinese people compared to western popularities, which might contribute to bias in data analysis.

Interestingly, ex-smokers, although having quit smoking for at least one year before surgery, had no significant advantage in survival compared to current smokers in the present study. However, this result should be cautiously explained. Several reasons might cause bias: (1) A small number of ex-smokers were defined in the present study; (2) Ex-smokers, as identified in the present study, had more multiple lesions and higher frequency of vascular invasion than non- and current smokers, both of which were well documented as risk factors of tumor recurrence and patient death[26]. The bias might dilute potential contribution of quitting smoking to RFS and LSM of patients, since it has been demonstrated that survival benefits might occur at least 10 years after quitting smoking[17]. Therefore, whether cessation of smoking preoperatively and for how long would have influence on postoperative prognosis need to be further evaluated. Cancer patients often undergo surgery shortly after diagnosis, making a planned cessation of smoking before surgery impossible. Due to possible increase of postoperative complications related to smoking, doctors would always suggest the patient quit smoking prior to surgery[11], [12].

Whether continuing cigarette smoking after surgery have any influence on tumor recurrence and patient survival has not yet been evaluated. One of the strength of the present study is that we further followed up the patients, and investigated the smoking status of them after recovery from the surgery. There were some patients continuing smoking within one year after disease treatment. The study demonstrated continued smoking postoperatively promoted tumor recurrence and exacerbated patient death. Therefore, even having no enough time for smoking abstinence before surgery, the patients should be strongly recommended to give up smoking after surgery.

The mechanism of cigarette smoking promoting HCC recurrence remains unknown. It has been demonstrated that smoking-related carcinogens could cause genetic and epigenetic alterations responsible for initiation and progression of cancer[27]–[29]. Several studies have proved unique molecular characteristics and behavior patters of adenocarcinoma of lung and colorectal cancer related to tobacco use[30], [31]. It is possible that smoking also influence tumor progression indirectly through altering host characteristics. Some experimental and clinical studies indicated that cigarette smoke aggravates liver injury and fibrosis by increased oxidative stress and production of pro-inflammatory cytokines, resulting in chronic liver disease progression[32]–[35]. Smoking might destroy host's immune defense system which is critical in preventing and clearing cancer cells[36]. Therefore, smoking promotes tumor progression possibly through smoking-associated genetic alterations, smoking-associated tissue damage fostering cancer cell spread, and immune depression.

In the present study, we found preoperative cigarette smoking is associated with worse RFS and increased LSM of HBV-related HCC at a possible dose-dependent manner. For smokers, continued smoking postoperatively might be strongly correlated with tumor recurrence and compromised survival. Therefore, smoking abstinence should be strongly recommended pre- and postoperatively. Future studies need to evaluate impacts of quitting smoking on HCC prognosis (how, when and for how long?), or to clarify possible changes of molecular characteristics of HCC related to tobacco use and whether these changes contribute to tumor recurrence.
